# Radiotherapy for oncologic emergencies on weekends: examining reasons for treatment and patterns of practice at a Canadian cancer centre

**DOI:** 10.3747/co.v16i4.352

**Published:** 2009-08

**Authors:** G. Mitera, A. Swaminath, S. Wong, P. Goh, S. Robson, E. Sinclair, C. Danjoux, E. Chow

**Affiliations:** * Departments of Radiation Therapy and Radiation Oncology, Odette Cancer Centre, Sunnybrook Health Sciences Centre, University of Toronto, Toronto, ON

**Keywords:** Emergency radiation treatment, metastases, spinal cord compression, weekend radiation treatment

## Abstract

**Purpose:**

Radiotherapy for oncologic emergencies is an important aspect of the management of cancer patients. These emergencies—which include malignant spinal cord compression, brain metastases, superior vena cava obstruction, and uncontrolled tumour hemorrhage —may require treatment outside of hospital hours, particularly on weekends and hospital holidays. To date, there remains no consensus among radiation oncologists regarding the indications and appropriateness of radiotherapy treatment on weekends, and treatment decisions remain largely subjective. The main aim of the present study was to document the incidence and indications for patients receiving emergency treatment on weekends or scheduled hospital holidays at a single institution. The secondary aim was to investigate the compliance of such treatment with the institution’s quality assurance policies, both local and provincial.

**Methods:**

From September 1, 2002, to September 30, 2004, patients being treated over weekends (defined as commencing at 6 pm on a Friday and concluding at 8 am of the next scheduled workday) and hospital holidays were retrospectively identified using the Oncology Patient Information System scheduling module. Relevant patient data—including patient age, sex, primary cancer site, specific radiation field, rationale for treatment, referring hospital, total treatment dose, radiation dose fractionation, inpatient or outpatient status, and duration of treatment—were collected and subsequently analyzed. Comparison to local policy was performed subjectively.

**Results:**

Over the 2-year period, 161 patients were prescribed urgent radiotherapy over a weekend or on a hospital holiday. Of this cohort, 68% were treated on both Saturday and Sunday, 22% on Saturday alone, and 10% on Sunday alone. Most patients presented with lung (31%), prostate (18%), and breast cancer (17%). The top reasons for referral for emergency weekend treatment included spinal cord compression (56%), brain metastases (15%), and superior vena cava obstruction (6%). Most of the indications for treatment generally followed the quality assurance policies implemented both locally and provincially.

**Conclusions:**

Patients treated over a weekend or on a hospital holiday were generally found to be treated with appropriate intent. Most treatment indications within this study both complied with provincial policy and showed a pattern of care similar to that seen in other studies in the literature. Local policy appears to be robust; however, policy improvements may allow for more cohesiveness across radiation oncologists in patterns of care in this important group of patients. Comparisons with practice at other institutions would be valuable and also a key step in developing sound guidelines for all members of the radiotherapy community to follow.

## 1. INTRODUCTION

Radiotherapy is an important treatment modality in the management of cancer patients. Approximately one half of all cancer patients require radiotherapy treatment during the course of their illness 1. Generally, treatment is accomplished using a system of low- and intermediate-priority planning and scheduling, and patients are treated daily during the working hours of the cancer centre. However, occasions arise on which radiotherapy must be planned and delivered on an emergency basis, and these occasions may occur outside of regular working hours. On weekends and statutory holidays, emergency treatments are subject to the discretion of the attending radiation oncologist.

Cancer Care Ontario (cco) defines oncologic emergencies as “medical conditions arising from a reversible threat to organ function requiring radiation treatment within a few hours of diagnosis.” The most common oncologic emergencies include malignant spinal cord compression, brain metastases (complicated by altered level of consciousness, uncontrolled seizures, or uncal herniation), superior vena cava obstruction (complicated by cardiovascular or neurologic compromise), acute airway obstruction, or uncontrolled tumour hemorrhage 1.

The Odette Cancer Centre (occ) in Toronto, Canada, offers radical and palliative radiotherapy treatment for cancer patients. The radiotherapy department at the occ regularly operates Monday– Friday, 8 am–6 pm 2. Additionally, a clinic dedicated to accommodating the urgent management of patients who require rapid access to radiotherapy for treatment of either their symptomatic metastatic or primary disease progression operates during working hours 3,4. The centre may operate on weekends and holidays to accommodate radiation treatment of oncologic emergencies involving patients for whom a delay in radiotherapy might compromise treatment outcome 2.

Standards for quality assurance are devised and implemented to facilitate commonality in establishing and evaluating quality assurance programs at cancer centres across Canada 1. For the purposes of the present study, quality assurance is defined as adherence of an institution to its own departmental policy. To date, literature to suggest appropriateness of delivering radiation treatment on weekends and holidays is scarce, and no cohesiveness is apparent in the standards of care for weekend radiation treatment 5. The main objective of the present study was to determine the incidence of radiotherapy treatment on weekends and statutory holidays, and the specific reasons for such treatment during the study period. The secondary objective was to ascertain whether the reasons for weekend and holiday treatment adhered to departmental policy and therefore achieved compliance with quality assurance procedures implemented by both the occ and cco.

## 2. MATERIALS AND METHODS

The radiation treatment policy for the department of radiation oncology at the occ states that weekend radiation treatments should be considered only in the following circumstances:

- When a delay in radiotherapy might compromise treatment outcome
- When pain control is impossible with medical therapy and, in the judgment of the attending radiation oncologist, radiation has the potential to provide pain relief within 48 hours

Treatment should not be undertaken on the weekend simply to facilitate the patient’s discharge from hospital unless the use of multiple fractions per day was not possible earlier in the week, or the inpatient bed is urgently needed for another oncology patient 2.

To handle weekend emergencies at the occ, a radiation oncologist and two radiation therapists are on call at all times during the weekend and on scheduled hospital holidays. A weekend is defined as commencing at 6 pm on a Friday and concluding at 8 am of the next scheduled workday (usually a Monday). Using the Oncology Patient Information System scheduling module at the occ, we retrospectively identified patients who were treated with radiotherapy on a Saturday, Sunday, or holiday between September 1, 2002, and September 31, 2004. These dates were chosen based on the retrospective availability of patient tracking into the radiotherapy system. Once the patients were identified, their identification numbers were used to obtain relevant information from the associated electronic patient records. Variables subsequently collected included patient age, sex, primary cancer site, specific radiation treatment field, rationale for treatment, referring hospital, attending radiation oncologist, total treatment dose, dose fractionation, occ inpatient or outpatient status, and duration of treatment. Results were then analyzed using descriptive statistics and, to assess adherence to quality assurance standards, were compared with the current departmental policy as described earlier for treatment of patients on weekends. All patient information was rendered anonymous, and research ethics approval was obtained.

Patient treatments were grouped into intervals based on the date of first visit to the radiotherapy department: September–December 2002, January– April 2003, May–August 2003, September–December 2003, January–April 2004, May–August 2004, and September 2004. The data were compared by interval and by year to account for inter-interval and inter-year variability.

## 3. RESULTS

From September 2002 to September 2004, 161 patients were treated as emergency cases over a weekend. In this patient cohort, 176 anatomic sites were planned for emergency radiation. The median number of patients treated was 21 per interval, with an average of 1–2 patients per weekend. Table I illustrates that, overall, most patients (68%) were treated on both Saturday and Sunday, less frequently (22%) on Saturday alone, and the least frequently on Sunday alone (10%). Additionally, 6.2% were treated on a statutory holiday attached to a weekend. (No data were collected for statutory standalone holidays not attached to weekends, and therefore no results can be generated for these unique situations.) The pattern was generally consistent over the 2 years and was not influenced by long weekends. The general trend as described was also consistent across intervals.

**FIGURE 1:**
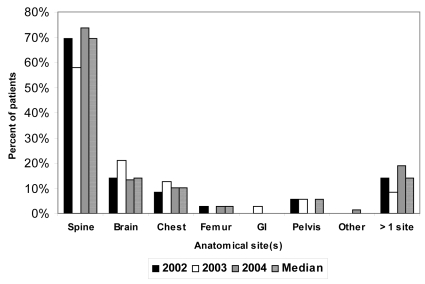
Anatomic sites treated as emergencies over a weekend or holiday. GI = gastrointestinal.

Table II lists the age, sex, primary cancer site, dose fractionation schedule, and inpatient or out-patient status of the studied patients. Median age was 67 years (range: 38–88 years). In 70% of cases, patients receiving treatment were outpatients; the remainder were inpatients either at the occ or a local community hospital. The highest frequencies of primary tumour diagnoses were lung (31%), prostate (18%), and breast cancer (17%). In 94% of cases, patients were treated with 20 Gy in 5 fractions. Of all cases treated over the weekend or on a hospital holiday, 66% received radiation to the spine; 16%, to the brain; and 11%, to the chest/mediastinum. In 15% of the overall population, radiation to more than one anatomic site was urgently prescribed (Figure 1).

Table III describes the main reasons for prescription of emergency weekend or holiday treatment. Spinal cord compression (56% of the cohort), brain metastases (both symptomatic and asymptomatic —15%), and superior vena cava obstruction (svco—6%) were the top three reasons for referral for emergency weekend or holiday treatment.

When reviewing treatments given over a weekend for compliance with departmental policy, a purely subjective analysis suggests that close to three quarters (129/169) of the reasons for treatment can be said to reflect appropriate intent. In a more detailed look at Table III, most reasons for treatment appear justified; however, reasons such as brain metastases, painful skeletal or spine metastases, and impending spinal cord compression were not considered justified under occ policy.

## 4. DISCUSSION

Approximately 5200 patients are seen annually for radiation oncology consultation at the occ. This number represents about 50%–60% of the local population of new cancer patients who will need radiotherapy during the treatment of their illness 1,6. Radiation for oncologic emergencies is quick and effective in alleviating symptoms caused by cancer progression. The success rate (at least partial symptom control) is approximately 70%–80% 7,8. The data extracted in the present study demonstrate that 1.6% of the entire annual patient population seen at the occ required radiation treatment on an urgent basis over a week-end or holiday 2. That result was expected because, of patients treated with radiation, only a very small population present with oncologic emergencies 8, and of those emergency patients, only 1 or 2 on average are treated over a weekend. When the total patient cohort of 161 in this study was averaged over the 2-year study period, the result was slightly more than 1 patient treated each weekend.

The overall number of cases treated with emergency intent on a weekend or holiday was not compared with the total number of emergency cases managed either during working hours or on weekday evenings, because these data were inaccessible. Consequently, it is difficult to ascertain if, relative to other patterns of care in this realm 5, more referrals were seen over the weekend than during the week. Whether the number of referrals for emergency treatment escalated on Friday as compared with other weekdays—as has been documented in past studies 9—was also not conclusive. However, over the 2-year period, we observed very few instances in which patients were not treated on weekends, suggesting that, although radiotherapy emergencies remain rare, they are indeed common enough to warrant sufficient resource allocation to maintain an appropriate standard of care for urgent symptomatic cancer patients.

Weekend treatment with radiotherapy is not a rarity in the management of curable cancer patients. Accelerated, continuous fractionation with 7-day-per-week courses has been studied and shown to provide excellent tumour control, particularly in head-and-neck cases 10. However, in the management of palliative patients, considerably fewer data are available on the suitability of weekend treatments and acceptable standards of care. A recent study into patterns of care demonstrated that the top three reasons for administering radiation urgently were myelocompression from spinal metastases, svco, and intracranial pressure from brain metastases 5. These appear also to be the most common emergency conditions across other series 7, and the data from the present study show similar correlation. It is well recognized that spinal cord compression and svco remain classified as “emergency” conditions, as does tumour obstruction or bleeding 11,12. These conditions are all documented within the cco guidelines—a situation that generally suggests that the intent for treatment in the present study complies with cco policy 1.

The occ departmental policy is different from that of cco: the department is bound by looser guidelines, without clear objectives for treatment on weekends and holidays. An example would be the statement “when a delay in radiotherapy might compromise treatment outcome” 2. With that statement in the policy, some radiation oncologists could justify treating an impending cord compression over the weekend if they feel it may compromise outcome; other oncologists may not feel the same way. The retrospective nature of the study made it difficult to determine from case records the exact intent for treatment in these more “grey” areas, and thus, became a limitation of the research. Certainly, treatment intent would be easier to document prospectively, because patterns of practice and adherence to policy can be clarified up front.

Although the occ policy remains generally suitable, it is indeed in need of some revision. But a difficulty remains: In the absence of published literature or practice guidelines to suggest the policies that should be followed, the decision to treat becomes a subjective one—at the discretion of the radiation oncologist. The notion of an “emergency condition” will definitely vary from one radiation oncologist to another. Aside from the top three, some of the indications for treatment are difficult to justify even with the small, but meaningful, percentage of patients who are prescribed treatment over a weekend when such prescription was unnecessary. Discrepancies will invariably arise between doctors in decision-making about emergency treatments—such as those relating to an impending spinal cord compression or to painful metastases with no neurologic or organ compromise, or even to new brain metastases in patients who are relatively asymptomatic and are controlled with medical management such as corticosteroids. Additionally, as seen in Figure 1, close to 20% of patients received treatment for more than 1 site over a weekend. Perhaps the need to expedite treatment for one urgent site allowed a second site that may not have been as great an emergency to be treated simultaneously—a situation that occurred in some of the cases in the present study. Along the same lines, it was unclear whether some treatments were delivered to inpatients over the weekend so as to expedite their discharge from hospital, as covered in the original occ policy. This information was very difficult to obtain, and again, the decision would be quite subjective and not necessarily documented.

The degree of symptom severity as perceived by the oncologist also plays a large role in deciding on weekend treatments, and perhaps the symptoms and severity that are classified as “urgent” (treatment within 72 hours) and “emergency” (same-day treatment) should be standardized within the occ. A questionnaire such as the Edmonton Symptom Assessment System could be a first-line tool to measure symptom severity and may be useful in this context to analyze and justify whether emergency treatment is warranted 13.

March 20, 2003, to May 31, 2003, represented the peak reported incidence of the severe acute respiratory syndrome (sars) outbreak in Toronto, during which increased infectious disease precautions were implemented. The occ was classified as a level 0 facility, where no probable or suspected sars cases were reported. However, the affiliated hospital, Sunnybrook and Women’s College Health Sciences Centre, was rated as a category 2 facility 14. Surprisingly, the patterns of care for prescribed emergency radiotherapy over weekends and hospital holidays remained unaffected based on the data tracked over the study period. Anecdotally, its was expected that, because the occ was identified at level 0, physicians would prescribe weekend radiotherapy with little hesitation, given that most patients (who were outpatients) could receive radiotherapy treatment without risk of exposure to sars. In fact, it might have been expected that the referral rate would decline, because the occ could not accept any patients transported from a facility with a status greater than level 1. The data did not reflect a decline; a similar pattern of care was documented over the entire 2-year study period (Table I).

To ensure that only oncologic emergencies as defined by the cco are treated outside regular hospital hours, our recommendation would include a revision to the current occ weekend treatment policy to explicitly state the conditions that qualify for weekend radiation treatment. These conditions would include malignant spinal cord compression, symptomatic brain metastases after initiation of medical therapy in patients who are unsuitable for surgery, superior vena cava obstruction (complicated by cardiovascular or respiratory compromise), acute airway obstruction, uncontrolled tumour hemorrhage, or neurologic compromise 1. Including the degree of symptom severity as measured by standardized questionnaires agreeable to the oncologists would also be useful. Collaborating with other radiation oncology centres in Ontario and Canada to document their patterns of care in the treatment of oncologic emergencies would certainly be ideal. Such collaboration would allow for improvement on the small number of cases currently analyzed and would initiate a more formal process, developing into practice guidelines in the future, to improve the standard of care for all patients who require emergency radiotherapy for their symptomatic illness.

Future directions would include prospectively collecting data on patients treated on weekends and all statutory holidays (whether attached to a weekend or standing alone), and expanding the search to include all emergencies treated at all hours, so as to make better direct comparisons between groups treated on the weekends and those treated at other times, and to document outcomes in a more effective manner.

## 5. CONCLUSIONS

Radiotherapy is a treatment modality necessary to address symptom control in cancer patients. The use of radiation for oncologic emergencies is justified, and weekend and holiday treatments are needed in a small percentage of patients. The present study confirmed that most weekend and hospital holiday treatments were intended for patients found to have an oncologic emergency, and that the treatments generally adhered to both departmental and provincial policy. However, there may be a place for revision and standardization of these policies to improve overall care for this small but important population of patients.

## Figures and Tables

**TABLE I:** Treatments delivered over a weekend or long weekend, September 2002 to September 2004

	Treatments delivered in															
	2002		2003						2004						Total	
	Sep–Dec		Jan–Apr		May–Aug		Sep–Dec		Jan–Apr		May–Aug		Sep–Dec (data for Sep only)		(*n*)	(%)
	(*n*)	(%)	(*n*)	(%)	(*n*)	(%)	(*n*)	(%)	(*n*)	(%)	(*n*)	(%)	(*n*)	(%)		
Saturday only	4	13	7	35	10	48	2	9	3	15	8	25	2	14	36	22
Sunday only	4	13	4	20	0	0	3	13	3	15	1	3	1	7	16	10
Both days	23	74	9	45	11	52	18	78	14	70	23	72	11	79	109	68
Total	31	100	20	100	21	100	23	100	20	100	32	100	14	100	161	100

**TABLE II:** Patient characteristics

Characteristic	2002	2003	2004	Overall
Patients treated (*n*)	31	64	66	161
Median age (years)	65	67	67	—
Sex [*n* (%)]				
Male	21 (68)	41 (65)	38 (57)	100 (62)
Female	10 (32)	23 (35)	28 (43)	61 (38)
Primary site [*n* (%)]				
Lung	15 (48)	20 (31)	15 (23)	50 (31)
Prostate	3 (10)	13 (20)	12 (18)	28 (17)
Breast	3 (10)	11 (17)	13 (20)	27 (17)
Lymphoma/myeloma	3 (10)	1 (2)	5 (8)	9 (6)
Colorectal	0 (0)	4 (6)	0 (0)	4 (2)
Others	6 (19)	7 (11)	14 (21)	27 (17)
Unknown	1 (3)	8 (13)	7 (11)	16 (10)
Ambulatory status [*n* (%)]				
Inpatient	12 (39)	17 (27)	20 (30)	49 (30)
Outpatient	19 (61)	46 (72)	46 (70)	111 (69)
Unknown	0 (0)	1 (1)	0 (0)	1 (1)

**TABLE III:** Reasons for emergency weekend radiotherapy

	2002		2003		2004		Overall	
	(*n*)	(%)	(*n*)	(%)	(*n*)	(%)	(*n*)	(%)
Spinal cord compression/cauda equina^a^	17	53	34	52	43	60	94	56
Brain metastases	4	13	11	17	10	14	25	15
Superior vena cava obstruction (svco)^a^	1	3	6	9	3	4	10	6
Acute nerve root compression/neurologic compromise^a^	1	3	3	5	5	7	9	5
Impending spinal cord compression	1	3	1	2	1	1	3	2
Non-compressive painful spinal metastases	4	13	0	0	4	6	8	5
Painful skeletal metastases (non-spinal)	1	3	0	0	3	4	4	2
Pelvic/gastrointestinal hemorrhage^a^	1	3	2	3	0	0	3	2
Acute airway obstruction (non-svco^a^)	1	3	3	5	1	1	5	3
Leptomeningeal disease^a^	1	3	3	5	0	0	4	2
Malignant bowel obstruction^a^	0	0	1	2	0	0	1	1
Increased symptoms from disease (non-obstructive)^a^	0	0	1	2	2	3	3	2
Total	32		65		72		169	

a Reason considered to be adherent to departmental policy at the Odette Cancer Centre^2^.
